# Incorporating new ways of doing by learning from everyday experiences and interactions using a multifactorial mHealth app

**DOI:** 10.1177/20552076221149293

**Published:** 2023-01-26

**Authors:** Emelie Mälstam, Ann-Helen Patomella, Eric Asaba

**Affiliations:** 1 Department of Neurobiology, Care Sciences and Society (NVS), Division of Occupational Therapy, Karolinska Institutet, Stockholm, Sweden; 2 Faculty of Health and Occupational Studies, Department of Public Health and Sport Science, University of Gävle, Gävle, Sweden; 3 Unit for Research, Development and Education, 531542 Stockholms Sjukhem Foundation, Stockholm, Sweden; 4 Graduate School of Health Sciences, 12944 Tokyo Metropolitan University, Tokyo, Japan

**Keywords:** digital health innovations, engaging occupation, blended health intervention, stroke prevention, mHealth, non-communicable diseases

## Abstract

**Background:**

Digital health innovations can support the prevention and management of risk factors for cardiovascular diseases, such as stroke. However, little is known about people's everyday experiences of digitally augmented stroke-prevention programmes combining onsite group sessions including peers and healthcare professionals with interaction and support from a multifactorial mHealth app.

**Objective:**

The aim of this study was to explore how people with stroke risk experienced interaction with a multifactorial mHealth app as support in the make my day stroke-prevention programme.

**Methods:**

Repeated interviews and observations with 12 adults with moderate to high stroke risk were analysed using a constant comparative method informed by constructive grounded theory.

**Results:**

Incorporating new ways of doing into everyday life involves a process through which participants learn from both being and doing in different environments (e.g., digital, physical and social). Digital self-monitoring combined with seemingly trivial everyday experiences played central roles in the process of increasing awareness of health and stroke risks, and providing tools to support increased self-reflection on everyday behaviours. Adoption of positive health behaviours in everyday life was supported or hindered by how easy to use and personally relevant the mHealth app was perceived to be.

**Conclusions:**

An experience-based group programme together with a personally relevant multifactorial mHealth app can be supportive in stroke prevention to increase general health literacy and stroke risk literacy, and promote the incorporation of new ways of doing in everyday life. Routines of doing digital self-monitoring and health-promoting activities were however strongly influenced by different environments in which choices are presented. It is therefore important to explore how both self-monitoring and health-promoting activities can be incorporated into everyday routines for different individuals. Research should also explore how personally relevant mHealth can be developed and integrated into prevention practices in primary healthcare.

## Introduction

Stroke is globally one of the leading causes of death and disability in people 50 years and older^1^ and is emerging as a major contributor to the global burden of disease in younger populations (ages 25–49).^2^ Stroke and other cardiovascular diseases (CVDs) have interrelated and coexisting lifestyle-related risk factors (e.g., smoking, physical inactivity, poor diet, elevated blood pressure, overweight and excessive alcohol consumption),^3^ which has informed the development of lifestyle interventions targeting more than one risk factor (i.e., multifactorial interventions). The diverseness of the stroke population, however, regarding the presentation of different combinations of risk factors for stroke, presents challenges for designing relevant preventative interventions. A cornerstone of and international priority for the sustainable prevention of many diseases caused by modifiable risk factors is the promotion of long-term adherence to an overall healthy lifestyle.^4^ In this article, a ‘healthy lifestyle’ includes addressing modifiable risk factors by promoting lifestyle habits important for CVD and stroke prevention^5,6^ and incorporating a pattern of self-chosen relevant activities (i.e., individual actions and behaviours) in everyday life that can contribute to health and well-being.^7,8^

As everyday life becomes more and more technology-based (e.g., due to mobile devices and Internet-based services) mobile phone health applications (mHealth apps) are increasingly used for managing chronic conditions, as well as promoting health and wellness.^9^ Apps can facilitate services that are available at any time and place and augment the usual care, with the potential to improve reach and access to services across various settings. Examples from CVD and stroke prevention show that such apps have a positive impact on CVD outcomes and individual risk factors for CVDs,^10,11^ as well as improving blood pressure control, increasing awareness of stroke and decreasing depression.^12^ Behavioural interventions among adult populations using digital self-monitoring have also shown potential to support self-management and promote health in an individual's daily life (e.g., improved medicine adherence, mood, health-related quality of life, eating habits, physical activity habits and decreased levels of pain and weight).^13^

Although there has been extensive research on the usability of various mHealth services for self-management and self-monitoring,^14–16^ it has been noted that much could be gained from exploring people's practical engagement with and incorporation of digital technology such as mHealth apps in everyday life.^17,18^ This knowledge is relevant because establishing and sustaining a healthy lifestyle – with or without support from mHealth – can be complex and challenging.^19,20^ A systematic review of blended interventions (e.g., that combine onsite sessions with augmented digital support) also reveals that only one or two main behaviours or risk factors (e.g., physical activity and nutrition) are usually targeted,^21^ while evidence-based guidelines for the primary and secondary prevention of stroke and other CVDs recommend targeting several risk factors simultaneously.^5,6^ Thus, there is a need to investigate how an intervention augmented with a multifactorial mHealth app can support long-term adherence to healthy lifestyle habits among people at risk for stroke.

In a newly developed digitally augmented prevention programme called Make My Day (MMD),^7^ a combination of multifactorial onsite group sessions with a multifactorial mHealth app is being tested in a Swedish primary healthcare setting as a model for non-pharmacological stroke prevention. The MMD prevention programme builds on the knowledge that people's health and well-being depend on everyday activities that are situated in a unique physical and social context. Participation in health-promoting everyday activities must therefore play a central role in prevention in order for it to be sustainable over time.^8^ Moreover, grounded in an understanding of humans’ need to be active and to have personally relevant and meaningful experiences,^22^ it is essential for people to be literate about health in relation to their own everyday life and to be actively involved in activities that fulfil their individual needs, in order for change to occur.^8,23^ The key behaviour change techniques (BCTs) in the MMD prevention programme – that is, the components designed to change behaviour^24^ – are therefore experiences with, and self-monitoring of everyday activities. More specifically, it focuses on performing and self-monitoring activities that are personally relevant, perceived as purposeful, infused with and promote positive feelings, and yield a sense of intense participation^25–27^ (i.e., engaging everyday activities, or EEAs).

A recent study of the MMD programme showed that utilizing the experience of EEAs in combination with digital self-monitoring holds promise, as health-promoting EEAs have been described as rewarding and as an origin of motivation for engagement in other health-promoting activities.^27^ Nevertheless, what a person considers to be an EEA is highly individual, may depend on the ‘right’ conditions (e.g., social context, surrounding environment or individual access to equipment),^25–27^ and can be viewed as positive, neutral or even negative in terms of individual health promotion.^28^ For example, EEAs have been described as work, social activities (e.g., doing something with friends and family) or various hobby activities that are done with others or alone (e.g., physical exercise, going for a walk with a camera). EEAs can also involve sedentary behaviours (e.g., online gaming, playing the guitar, or reading books), which is why MMD explores the redesigning of some EEAs to involve less sedentary time.^7^

Utilizing digital technology to register and engage in ‘a broader perspective’ of health that involves one's activity patterns in everyday life and focuses on meaningful activities and lifestyle goals has been described as a greatly needed dimension for some people, to achieve changes in everyday life.^29^ A central part of MMD, therefore, involves identifying and following up on individual lifestyle goals that are based on the participants’ EEAs; in this way, MMD takes a slightly different approach than other lifestyle interventions involving more commonly used BCTs.^30^ In addition to the usual self-tracking of, for example, physical exercise, MMD includes the self-monitoring of broader aspects in everyday life that are important for health (e.g., EEAs, perceived wellbeing, satisfaction, sleep and psychosocial stress).

Lifestyle redesign studies in which the key BCT is participation in health-promoting activities have reported a significant positive impact on social participation, along with improvements in mobility, vitality, mental health, overall life satisfaction and well-being among well elderly people.^31–34^ Other findings include improved clinical CVD outcomes in people with diabetes, people at risk for CVDs and people with an already established CVD.^35,36^ To the best of our knowledge, however, no studies have explored digitally augmented stroke-prevention programmes that combine personal attention and support from health professionals and peers in a multifactorial onsite group intervention with a multifactorial mHealth app to stimulate people to enhance their literacy and participation in individually relevant engaging and health-promoting activities for early disease prevention. Knowledge of people's experiences with such support is therefore lacking.

The aim of the present study was to explore how people at risk of stroke experienced their interaction with a multifactorial mHealth app as support during and after the MMD stroke-prevention programme.

## Materials and methods

Constructivist grounded theory^37^ informed the study design and analysis. With its focus on systematically exploring views and perspectives on social processes and circumstantial influences on patterns of behaviours, iteratively, this approach is well suited for this study.

### Study setting and the MMD programme

**Figure 1.**
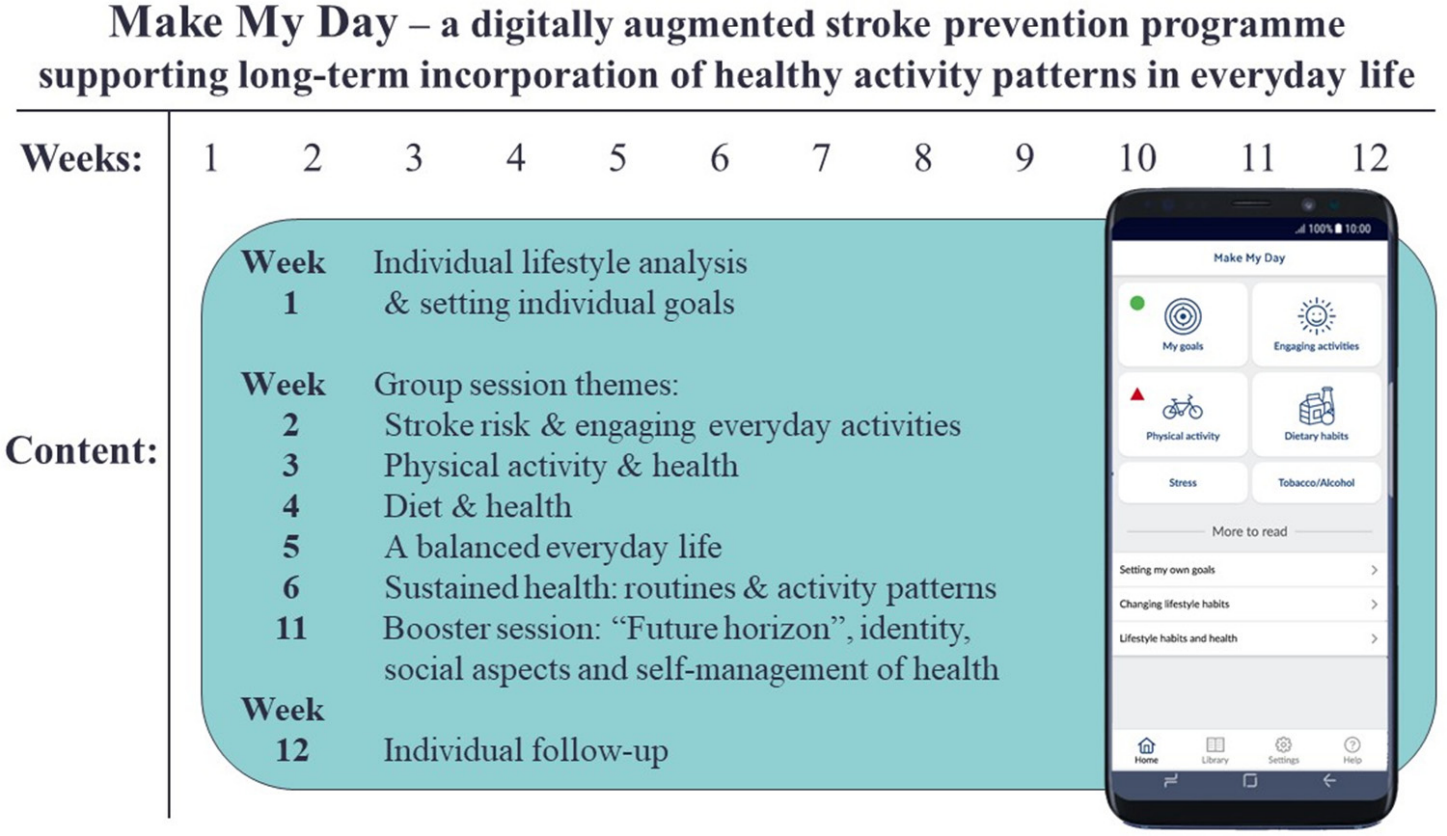
The MMD prevention programme flow chart.

This study is part of a larger research project testing and exploring MMD, a non-pharmacological, mHealth-supported, multifactorial and person-centred stroke-prevention programme.^7^ As shown in Figure 1, MMD combines the utilization of an individual lifestyle analysis, a multifactorial mHealth app, and onsite group sessions. The overall aim of these combined methods is to promote the incorporation of healthy and sustainable activity patterns in everyday life among people at risk for stroke.

During the individual meeting and lifestyle analysis, the participants work one-on-one with a health professional to conduct a screening of the individual's background, stroke risk and current life situation. With support from the Canadian Occupational Performance Measure (COPM),^38^ the participants define and prioritize areas in their everyday life that are both problematic and important in relation to their health and wellbeing. Next, they formulate personally meaningful and relevant lifestyle-related goals and self-assess their current performance and satisfaction with these goals. During their participation in MMD, the participants register and self-monitor their lifestyle habit goals using the MMD mHealth app. After their participation in MMD ends and for up to 12 months afterwards, their goals are followed up using COPM. The individual lifestyle analysis and the use of COPM for setting goals serves as the basis for the person-centeredness in MMD and as a start for individual reflection on one's activities and habits in everyday life and their connection to health and wellbeing.

During the onsite group sessions, the participants interact with peers and healthcare professionals as they learn about stroke risks, EEAs, healthy activity patterns and healthy lifestyle habits, and try out health-promoting activities together in the group. The MMD mHealth app is integrated as part of the prevention programme and is used from the start, augmenting the physical onsite group sessions with digital self-monitoring. Participants do registrations in six modules in the app on a daily basis (e.g., lifestyle goals, EEAs, physical activity, nutrition, stress, and tobacco and alcohol). The ‘physical activity’ module for example involves registrations of the number of steps and various physical activities performed within a day (e.g., exercise, physical activity, sleep, sedentary time and remaining time). Nutritional registrations are moreover deliberately focused on aggregated information (e.g., fist-size portions of fruit and vegetables) based on recommendations from the Swedish Food Agency and Nordic National Recommendations on health-promoting nutritional intake.

**Figure 2.**
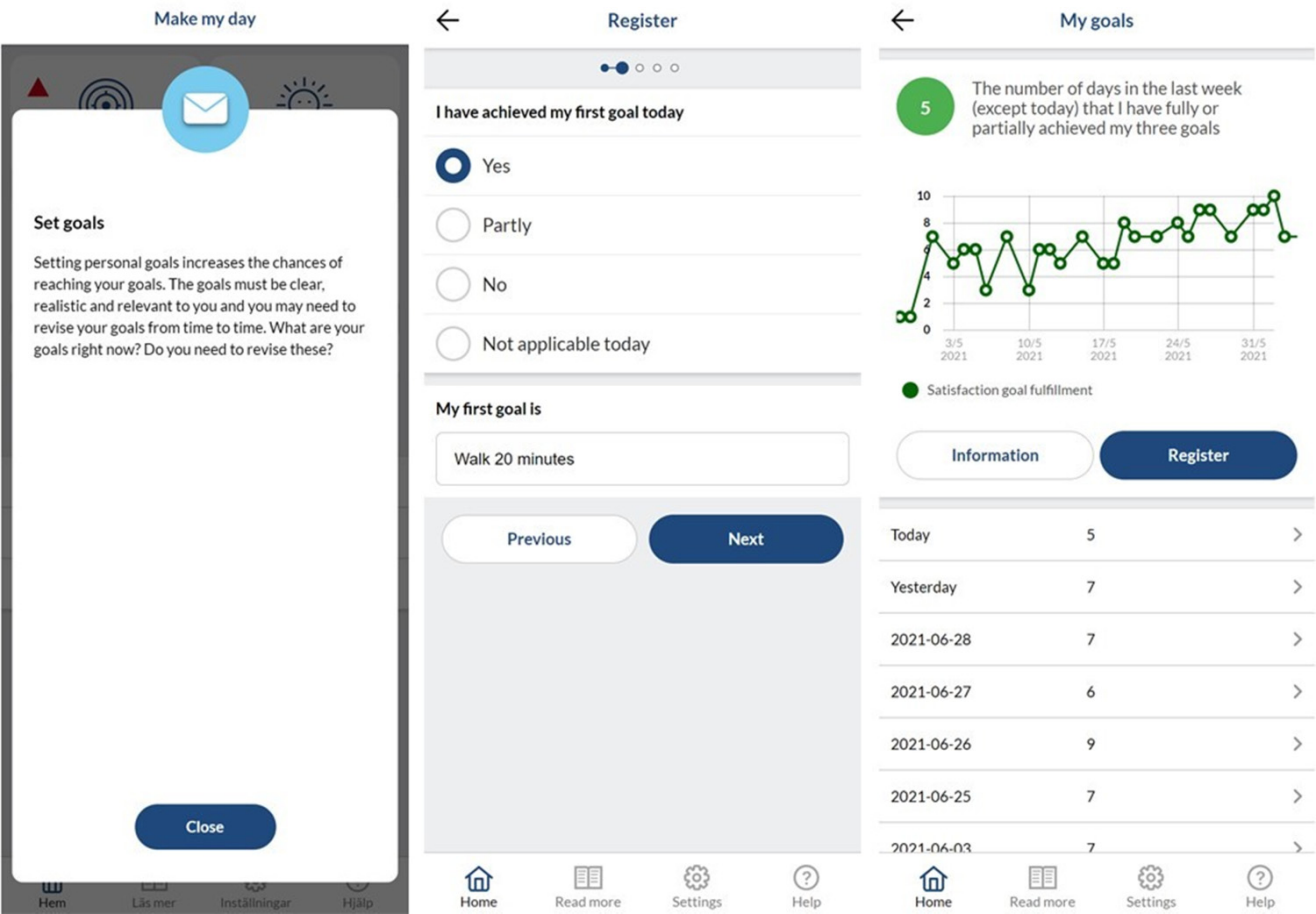
Examples of a push message, registration and generated statistics in the MMD mHealth app.

The app generates statistical feedback from registrations and provides generic supportive push messages following each weekly onsite session (Figure 2). The push messages were meant to remind the participants of weekly themes and motivate them to implement behaviour changes. The advantage of the digital augmentation of the programme is that it provides a tool that is available 24/7 to support people in changing and maintaining behaviours that are important for their individual health and well-being. The MMD mHealth app, referred to herein as ‘the app’, was produced in collaboration with ScientificMed Tech AB (now part of Cuviva AB).

### Study sampling and participants

Participants in the present study were recruited from a case study (*n* = 6) and from the intervention arm (*n* = 14) of a randomised controlled pilot study (*n* = 29) testing the MMD programme. Individuals at risk for stroke that were enrolled in three different MMD intervention groups were invited by telephone to participate in this study, using convenience sampling.^39^ Inclusion criteria for participation were: (a) age of 45–75 years; (b) the presence of three or more stroke risk factors scored as moderate to high risk in a stroke risk scorecard^40,41^ (including the following risks: smoking, physical inactivity, poor diet/overweight, diabetes, high blood pressure, atrial fibrillation, high cholesterol and family history); (c) access to a smartphone or wireless device; and (d) motivation for lifestyle change. Exclusion criteria were: (a) ongoing drug abuse; (b) history of a previous stroke; and (c) non-Swedish language fluency, since the app prototype was only available in Swedish. In total, 13 participants were invited and 12 agreed to participate.

The participants consisted of six men and six women aged 49–70 years with a mean age of 59 years, all living in the city or suburbs of Stockholm, Sweden (see Table 1 for the participant information). Two participants had origins outside of Sweden. The educational level varied, with one participant having primary education, five having secondary education and six having higher education. Three participants had a low income (<19,300 Euro yearly), seven had a middle income (>19,300 <58,000 Euro yearly) and two had a high income (>58,000 Euro yearly). All participants had access to either a smartphone or other wireless device, and all participants but one were frequent users of smart phone technology in everyday life. Eight participants considered that they had the skills needed to use smart phone technology and different health apps, while four participants did not. None of the participants had had a previous stroke.

**Table 1.** Participant information.

Person	Sex	Living situation	Number of medium to high stroke-risk factors*
1	Female	Living alone	3
2	Female	Living together	3
3	Male	Living together	3 **
4	Male	Living together	4
5	Female	Living alone	4 **
6	Female	Living alone	4 **
7	Female	Living together	4
8	Male	Living together	4
9	Female	Living together	5
10	Male	Living together	5 **
11	Male	Living alone	5 **
12	Male	Living together	6 **

* according to a stroke risk score card including the following stroke risk factors: blood pressure (>120–139/80–89), atrial fibrillation, smoking (smoker or trying to quit), cholesterol (>200), exercise (<150 min per week), self-reported and objectively measured to be overweight, and hereditary stroke.

** including diagnosis with a previous TIA, considered as a high stroke-risk factor.

Participation in this study was based on both written and oral informed consent. The process of obtaining informed consent was undertaken at several time points as this study was conducted over 18 months’ time. Firstly, written and oral informed consent was obtained when participants were enrolled in the studies testing the MMD intervention and thereafter also when the participants were asked to participate in follow-up interviews and observations about their participation in the MMD. Where follow-up interviews were conducted with the same participant, informed consent was again confirmed orally. The ethics review for this project was approved by the Regional Ethical Review Board in Stockholm, Sweden (Ref. No. 2015/834–31, 2016/2203–32, 2019–01444, 2020–03822).

### Data gathering

All participants initially shared their background information during their first screening and individual lifestyle analysis. Thereafter followed semi-structured interviews^42^ and observations.^43^ Data for the study was collected over 18 months (2018–2020). Participants at risk for stroke with a previous Transient Ischaemic Attack (TIA) were interviewed individually three times over the course of a year. These interviews covered experiences of participation in MMD and experiences of behavioural change while using health apps in general and the MMD mHealth app in particular. Each interview was built on the previous one, and the range of topics was subsequently narrowed down to answer analytical questions or fill conceptual gaps.^37^ Six participants with moderate to high risk for stroke (but without TIA) were then interviewed once. These participants were first asked about their experiences and objectives in using digital technology in everyday life and, more specifically, in using health apps and the MMD mHealth app. The participants were then observed while using the MMD mHealth app. During these observations, the participants were asked to verbalize their thoughts to elicit real-time actions and descriptions of experiences relating to lifestyle habit change while engaging in the app.^43^ Participants were encouraged to say whatever came into their mind and were prompted with questions such as: ‘What are you looking at and/or doing in the app?’, ‘Why are you doing that?’ and ‘What are you thinking of when using the app?’.

All interviews and observations were audio-recorded and conducted in quiet locations agreed upon with the participant. Reflective notes were written in tandem with the interviews and observations describing the interviews and utilization of the app.

### Data analysis

Exploration of the data, including initial and focused coding, constant comparisons and memo writing, were conducted as described by Charmaz^37^ and covered all audio-recordings, which were transcribed verbatim, as well as reflective notes from the interviews and observations. During the initial coding, line-by-line coding was conducted while remaining close to the original text. Example of initial codes included: *Keeping track*, *Seeing what one does in the app*, *Reflecting about what one does*, *Realizing when one is snacking*, *Like getting a receipt*, and *The importance of smart feedback*. Focused coding was then applied to synthesize and explain data segments. Examples of focused codes included: *Transacting information on experiences, Visuals as inspiration and nudges for healthy behaviours, Individual and smart design to support habit changes*, and *Registrations and self-reflection as support for doing*. Codes were continuously and systematically compared – both within and between interviews and observations – and sorted into categories. The analysis process was supported by memo writing about significant and frequent codes. Some of the most frequent codes were: *A tool for visualizing and self-analysis, Confirmation from what the app shows*, *Increased literacy through reflection*, and *A need to adapt to the individual.*

Constant comparisons and pre-understandings and our roles as researchers versus interventionists were discussed in reflective group meetings throughout the analysis. Consolidated criteria for reporting qualitative research (COREQ)^44^ were utilized to report this qualitative work (see supplementary material for study descriptions and details of authors background). Atlas.ti software was used to organize the data. By synthesizing all the categories, one core category was identified.

## Results

In the result section one core category *Incorporating new ways of doing by learning from everyday experiences* is presented, followed by three underpinning categories (1) *visualizing health and change through registrations and self-reflection*; (2) *making healthy choices with personally relevant support*; (3) *developing self-monitoring routines to hold onto a healthy lifestyle.*

### Incorporating new ways of doing by learning from everyday experiences

The core category, *Incorporating new ways of doing by learning from everyday experiences*, refers to a process through which participants learned from both being and doing in different environments (e.g., digital, physical and social). The term ‘doing’ in the results reflects action and experientially unique engagement in a particular activity that is integrated into everyday life. When used in a context, such as ‘doing walking’, doing represents a unique experience and active engagement in this particular activity, which is differentiated from the person taking a walk without the deliberate and active choices that imbue the term ‘doing’ here.

**Figure 3.**
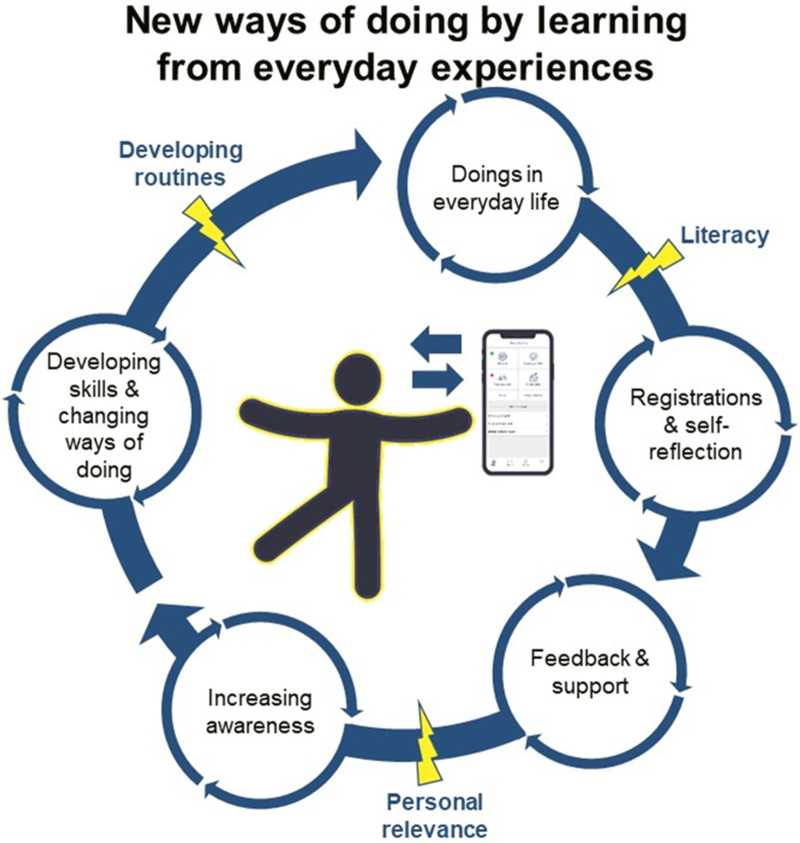
Illustration of the core category and significant aspects in the participants’ process towards new ways of doing; lightning bolts represent hindering or facilitating factors.

As displayed in an illustration of the findings (Figure 3), digital self-monitoring and seemingly trivial everyday experiences played central roles in the participants’ learning process by providing tools to support increased self-reflection on everyday activities and behaviours and on how these affected the participants’ lifestyle habits and overall health. Iterations of actively engaging in everyday life and interacting with the app were found to contribute to deeper health literacy involving health knowledge, health awareness and the adoption of healthy behaviours (e.g., walking, exercising, eating fruits and vegetables and reducing smoking) in everyday life.

The participants’ everyday experiences varied widely, ranging from short experiences (e.g., reading the ingredients list on a loaf of bread at the supermarket) to more comprehensive and complex experiences (e.g., participating in activities in the prevention programme and discussing experiences with peers in the intervention). These very different experiences had one thing in common: an inherent possibility of increasing health awareness and the adoption of healthy behaviours if the participants were simultaneously self-monitoring in the app. This possibility could be facilitated or hindered depending on whether the participants did or did not develop routines of doing engaging and health-promoting activities while self-monitoring regularly. The participants’ level of literacy regarding both health and digital technology and the personal relevance of the features in the app were factors in speeding up or slowing down the participants’ iterative process of incorporating new ways of doing in everyday life. The participants’ experiences underpinning the core category are presented with quotations in the following categories.

#### Visualizing health and change through registrations and self-reflection

By exchanging information with the app (e.g., doing registrations and receiving automatically compiled feedback in the form of statistics) on their patterns of activities and experiences over a week, the participants increased their awareness and knowledge of what they were actually doing and how this connected to their habits and health. What had been experienced as abstract prior to using the app became more tangible as the results became visual after continuous registrations. One of the participants described how the app had been a particular support in terms of her eating habits:

I always reflect on it [her lifestyle]. I would have done that even without the app. But it has been a real support … especially with regard to mealtime habits … I actually eat sweets more often than I think. Those little bar graphs. It is really good, actually, that I saw that. Because, somehow, one lies to oneself, apparently. (Participant 1)

In regards to learning and incorporating new doings, the participants expressed that doing activities (whether health promoting or health compromising) in tandem with daily registrations allowed them to discover their own strengths and weaknesses and their current patterns of activities and habits, while keeping track of risk factors. The participants described this process as something that triggered visions of their future lifestyle and health. Interaction with the app was likened to a way of mirroring their behaviours and habits, and repeated registrations of their doings allowed participants to imagine themselves and their overall lifestyle, which was important in reaching their goals. One participant commented:

Being able to visualize is really important. The app is a really good tool for that … to achieve goals, you must be able to visualize, and to visualize, you must get this input [results or statistics from the app] all the time. Either you have a book and write it down, or an app that pops up a few times a day … it is about seeing yourself. (Participant 8)

Personal relevance – such as preferences and previous life experiences – and the personal tailoring of content and approaches in the app were described as important:

For an app to work, it must be adapted to the person who will use it in a different way, so that it will be … we all have different ‘carrots’ [incentives]. Do you want the whip, encouragement, or what do you want? (Participant 7)

Some participants favoured seeing themselves through detailed statistical feedback, while other participants appreciated feedback through text messages or illustrations. One participant, who had prior experience with an eating disorder that was associated with control over specific measures, described the importance of not becoming too focused on specific measurements. This participant reasoned that, in order for the app to be useful in helping her move towards a healthy lifestyle, it was important for it to be fun, engaging and focused on an overview of health, rather than being characterized by control and punishment. In contrast, other participants with other experiences and preferences preferred seeing more detailed numbers and trends and having control; for them, having a risk perspective pushed them to take action on sedentary behaviours or eating enough fruits and vegetables.

#### Making healthy choices with personally relevant support

Participation in the onsite group sessions and interaction with the app were described as prompting the participants to take action on certain health-compromising behaviours (e.g., snacking, sitting still, smoking and excess stress):

Technology is absolutely incredible … it's that you get a receipt [confirmation] … if you don’t have an app like this … and no one comes and says, ‘what a good job you did’. This [feedback from the app] is like a pat on the shoulder … It's like a receipt … and that … motivates me. (Participant 4)

However, the app was especially valued for its continuous confirmation of what the participants were actually doing in everyday life, which was not as evident without the app. The individually compiled statistics in the app (e.g., bar charts and graphs) provided confirmation of whether the participants were headed in the right direction towards their lifestyle goals. Incorporation of healthy choices in everyday life happened a little at a time, both by receiving confirmation from the app and by learning from different everyday experiences in digital, physical and social environments. Actively utilizing the app's feedback and results and continuously being reminded of individual goals in the app cued the participants to enact healthy behaviours:

It [the app] reminds you every day when you open it … the goals that one has set, all the thoughts that one has written down, and then one gets … these kinds of cues. (Participant 8)

The participants knowingly and unknowingly tried to change and incorporate new ways of doing into their everyday life while exchanging information with the app. For instance, one participant became more aware of what she wanted to do more of in everyday life (e.g., engage in meaningful activities, stress less and eat healthier). As another example, participants described how registering steps in the app helped them take up and sustain old routines (e.g., evening walks). The participants reasoned that it was important for the experience of self-monitoring to be fun and engaging in order to be motivating. Therefore, it would have been helpful for the app to correspond to the participants’ preferences as well as their digital skills, including both numeric and visual literacy (e.g., reading graphs and interpreting numbers). The participants also noted a missing feature in the app: there was no overall picture of the progress made towards decreasing the risk of stroke. The addition of this type of visual imagery was suggested as a way to push the participants to make and sustain changes:

It should be, that it gets summed up somewhere … your progress, in terms of risks for stroke … It would give me good awareness of what I am doing … about how I live my life. (Participant 7)

Another participant found that it took time to understand what her EEAs were and how they were connected to her health and risks. She reasoned that, now that she had learnt more from both self-monitoring and participating in EEAs, it would have been fun to be able to continue self-monitoring and reflecting on EEAs, to support her in continuing to participate in these engaging activities.

The app, with its different modules, was considered to be helpful for making healthy choices, as it was an easily available tool that reminded and prompted the participants in everyday life. Yet the simultaneous experiences from the onsite group sessions were important, as they also provided peer support, prompts and encouragement for making healthy choices.

#### Developing self-monitoring routines to hold on to a healthy lifestyle

The participants found it important to develop and sustain self-monitoring routines in order to incorporate new ways of doing in everyday life. Some participants had a hard time developing a routine of doing registrations that was incorporated within other daily activities, while others had developed a routine of always doing their registrations at a specific time and space; the kitchen table after dinner while reflecting on the day alone or with a spouse, or doing registrations and reflecting when watching TV or when going to bed. One participant stated:

… in front of the TV. The last thing one does. Then I sit and play around with all that stuff … plan … fill in everything … I think it's convenient … Then you learn … You have to do it [register in the app] continuously … Then you get an overview of this, and it is good to remind oneself all the time that one has this knowledge… (Participant 2)

The participants found that having a routine for documenting and reflecting on everyday experiences supported their continued engagement in health-promoting habits and using the app. Habits and registrations went hand in hand:

Since I started with this [the prevention programme and using the app], I have started to eat at least one fruit and one raw vegetable almost every day. (Participant 4)

If their routine of doing registrations and reflecting, in tandem with engaging in different activities, was not developed and sustained, the participants found that their learning process and their engagement with the app slowed down or were disrupted. When participants lacked a routine and context for self-monitoring, they experienced the registrations as less meaningful or motivating.

When the participants continued doing self-monitoring of everyday experiences and health after the completed intervention, many continued to use digital tools (e.g., health apps, smart watches, wearables or documents on a computer). A couple of participants went back, or continued, to use analogue tools as well (e.g., a physical notebook or calendar). The participants’ preferences were often rooted in previous experiences of doing self-monitoring and in current life situations (e.g., work and family situation, being pressed for time), which affected how they wanted to continue self-monitoring. To continue or change their routine of doing self-monitoring from analogue into digital self-monitoring, the participants found it important for the digital tools to be easy yet smart, so they wouldn’t get tired of them. As one participant said:

…I get bored. I think it's interesting for a while … at the same time, it's good for me to stay on top of things. I should eat the right things … that's good. I’m not saying it's bad. But I haven’t gotten the right feel for it [the MMD mHealth app]. (Participant 11)

The other participants reasoned in the same way: they wanted the app to be smarter and also to continuously evolve, so it would keep being engaging and promoting the continuation of a self-monitoring routine. Just doing registrations (e.g., goals, food intake, exercise, stress, sleep and engaging activities) was found to be a very positive routine to have and reflect on. However, it was necessary to balance the number of things the participants needed to include in the routine of self-monitoring. Some of the features in the app were not automatized (e.g., steps), which risked decreasing participants’ motivation for keeping up their self-monitoring routine. It was important for the utilized technology to suit the participants’ preferences and needs, in order for it to motivate them to continue doing self-monitoring. Participants who had developed a daily routine of summarizing their activities and experiences felt more supported in creating opportunities to consolidate certain health-protective behaviours in everyday life than those who had difficulties developing a self-monitoring routine.

## Discussion

The aim of this study was to explore how people at risk of stroke experienced their interaction with a multifactorial mHealth app as support during and after the MMD stroke-prevention programme. The findings indicate that, if mHealth is situated in personally relevant contexts and routines, it can be a useful tool in supporting self-monitoring, self-reflection, and participation in EEAs during an intervention. Undergoing situated everyday experiences with the app was central to the participants’ learning, and was experienced over time as a support in changing several health-related behaviours. For instance, tracking physical activity in relation to health and EEA-based lifestyle goals resulted in the participants receiving visual and numeric feedback in the mHealth app. The routine of logging information (self-monitoring) resulted in opportunities for reflection (self-reflection), which sometimes led to changes being made in everyday habits – a finding that has also been reported elsewhere.^45^ Participants doing of EEAs, however, were strongly influenced by different physical, social, and digital environments, in which different choices were presented.^46^

In order to support behavioural change, it has been reported to be desirable for health apps to focus on self-monitoring of broader lifestyle goals, such as participation in EEAs,^27,30^ in contrast to the more detailed self-tracking of numbers (quantification of self), which works for some people but not for others.^17,18^ Yet, studies with this perspective are scarce. In addition, this study also suggests that the newly introduced routine of registering data regarding EEAs in the app was more than self-monitoring: the registering/feedback aspect of mHealth in combination with having an experience of an activity fresh in mind, ‘nudged’ the participants to do things differently. ‘Nudging’ is a concept that describes how even minor changes in people's environments can influence the outcome of people's decisions.^47^ In nudging theory, nudges typically occur without the decision-maker noticing the influence. In the present study, some participants were unknowingly influenced in their decision-making and health-related behaviours, while others described being more conscious of nudges and deliberately trying to use them to change their lifestyle habits.

Nudges have also been called ‘teachable moments’,^48^ which resonates well with the results from this study in which the participants described seeing their risks more clearly visualized in the app, learning by doing and by having different experiences in varying environments (e.g., digital, physical or social). Learning through experience or ‘learning by doing’ is a well-established concept that is widely used in education.^49^ Other preventative interventions have drawn on learning-by-doing concepts to support engagement and meaningful activities among the well elderly.^31–36^ In non-pharmacological stroke-prevention and digitally augmented multifactorial lifestyle interventions, experiential approaches or nudging have not been significantly utilized to promote healthy activity patterns in everyday life. The participants valued and positively experienced MMD's combination of approaches, which involved both physical/social and digital features; this finding indicates the importance of studying and evaluating the feasibility and effect of using such approaches further in the prevention of stroke and other lifestyle-related diseases.

By routinely registering and participating in health-promoting EEAs, the participants were motivated to engage in health-protective behaviours and to pursue lifestyle-related goals. This result strengthens findings from previous studies exploring the feasibility of MMD for promoting healthy activity patterns in everyday life.^27,50^ However, the importance of developing a routine of self-monitoring in combination with participation in EEAs was key in the iterative behavioural change process and in actually using the app. To facilitate the ‘just-right fit’ of the app, and nudges, and to assist people in integrating both self-monitoring routines and EEAs in their activity patterns, it is important to continue creating person-centred designs with integrated personally relevant feedback in future digital health innovations. A lack of tailored and timely delivered digital nudges may otherwise limit participants in their use of the app.^46^ In future app development it will be valuable to consider a range of psychosocial, emotional, behavioural and/or contextual circumstances.^51^

As personalization has been described as crucial, a digital environment must align with the individual's needs to be effective.^52,53^ In the case studied here, using COPM^38^ to help participants set personally relevant lifestyle goals at the start may have contributed to a personalized experience when using the app. In the future, it is however important to further tailor the app content to peoples’ literacy levels (e.g., numerical, visual and health literacy) to provide incentives for completing registrations and support reflection. Gamifying features can be one way to motivate app users to be more consistent and precise with self-monitoring.^54,55^ More importantly in this study, something that the participants were missing in the MMD app, which is recommended to be incorporated in the next phase of app development, was to both receive information and visuals portraying the individuals stroke risk evaluation over time, as this was something the participants described as a potential support for behaviour change and maintenance. The results also revealed a need for automatizing more features in the app (e.g., steps, physical exercise, physical activity and sleep). The participants described a certain degree of time burden when registering their daily data log. Still, the logging of data enabled new routines and may have triggered reflection and even a consideration of alternatives.

Future studies are needed to explore how mHealth can be integrated into everyday routines and activity patterns. Moreover, the idea of incorporating self-monitoring routines in people's everyday life through gamification should be investigated further. Even if personally adaptable apps can technically be developed, it is unclear whether Swedish primary healthcare has the resources to do so when providing a personalized app for early stroke prevention. App engagement and adherence rates, extending from study attrition rates, are also important additional pieces to understand how the MMD mHealth app support behaviour change. This data has been collected and will be presented in future studies focusing on the acceptability and feasibility of the intervention.

Lastly, in this study, the participants’ use of the app was not restricted by a lack of Internet and cell coverage, and all the participants had access to smart phones. However, many parts of the world do not have the necessary civic infrastructure and wide availability of smart phones experienced in this study, which would hinder the applicability of this research. Smart phones have been used in interventions in diverse global contexts^10–13,15,16,19,20^ and within low-income countries with some success.^14,56^ Nevertheless, the purchase or availability of smart phones and access to apps can be prohibitive in socio-economically vulnerable areas. Moreover, people with little experience using a smart phone or with functional impairments that make it difficult to navigate on a smart phone are likely to experience difficulties using such an app.^57^

### Strengths and limitations

This study contributes to the development and evaluation of digital health innovations,^53^ a key part of which involves qualitatively exploring aspects of app interactivity connected to the process of changing behaviours. The results provide an early exploration of how people at risk for stroke experience and utilize app interactivity in combination with doing EEAs as a way to make changes towards a healthy lifestyle. This is the first study to examine this topic, probably because this type of mHealth-supported multimodal intervention is new, and aligns with current guidelines.^5,6^

The approach taken in this study is based on constructive grounded theory,^37^ which made it possible to capture the interaction and contextual influences needed to understand people's experiences with app interactivity connected to the process of behavioural change. A systematic, qualitative method was well suited to the aim of this study, as it endorsed an iterative and open-ended approach to data collection and analysis.

The sampling approach, which included participants at different time periods (during, after and through later follow-up after the MMD prevention programme had ended) and from healthcare units in different geographical locations increased the heterogeneity in the participants’ experiences. However, the study has some limitations: the app was not translated from Swedish into other languages, which will be an important future development to allow more people to test MMD in a variety of settings and societies. The study lacks input from people without access to technology, with cognitive or functional impairments, or with less favourable socio-economic conditions. A larger sample may also reveal more varied preferences or capacities for using apps to monitor stroke risk and activity patterns. When interpreting the findings, therefore, it should be noted that the generated data in this study are situated in a specific time, place and culture, and should be understood within the particular context of the study setting, the intervention and the studied sample.^37^

Combining observations with follow-up interviews over a longer period of time provided rich data that strengthened this study's credibility. Continuous reflective analysis meetings within the research group and the use of a combination of methods provided triangulation and enhanced the reflexivity of the research process.^58^

### Conclusions

Utilizing an experienced-based approach that combines participation in EEAs with personally relevant digital augmentation holds potential for addressing several lifestyle-related stroke risks simultaneously and promoting the incorporation of new ways of doing in everyday life. Such multifactorial lifestyle interventions allow people to have digital, physical and social experiences while self-monitoring their EEAs and lifestyle habits. In this way, people can increase their health awareness while being supported by nudges, triggering reflection and consideration of alternatives. Routines of doing EEAs and digital self-monitoring were however strongly influenced by the individuals different conditions, circumstances, and environments. Future studies should therefore continue to explore how EEAs and self-monitoring can be incorporated into everyday routines and activity patterns for individuals in different contexts with different conditions, and how personally relevant mHealth can be developed and integrated into prevention practices in primary healthcare.

## Supplemental Material

sj-docx-1-dhj-10.1177_20552076221149293 - Supplemental material for Incorporating new ways of doing by learning from everyday experiences and interactions using a multifactorial mHealth app Click here for additional data file. Supplemental material, sj-docx-1-dhj-10.1177_20552076221149293 for Incorporating new ways of doing by learning from everyday experiences and interactions using a multifactorial mHealth app by Emelie Mälstam, Ann-Helen Patomella and Eric Asaba in Digital Health
