# Supplementary material for: Incorporating new ways of doing by learning from everyday experiences and interactions using a multifactorial mHealth app
Source: Digit Health. 2023 Jan 26;9:20552076221149293. doi: 10.1177/20552076221149293 (PMC9903038; doi:10.1177/20552076221149293)
Supplement: sj-docx-1-dhj-10.1177_20552076221149293 - Supplemental material for Incorporating new ways of doing by learning from everyday experiences and interactions using a multifactorial mHealth app [file sj-docx-1-dhj-10.1177_20552076221149293.docx]

**Supplementary Material according to the Consolidated criteria for reporting qualitative research (COREQ) [1]**

| **No** | **Item** | **Description** |
| --- | --- | --- |
| **Domain 1: Research team and reflexivity** | | |
| *Personal characteristics* | | |
| 1. | Facilitator | Emelie Mälstam, Ann-Helen Patomella, Eric Asaba |
| 2. | Credentials | Emelie Mälstam (MSc), Ann-Helen Patomella (Prof. Dr.), Eric Asaba (Prof. Dr.) |
| 3. | Occupation | EM: PhD student; AHP, EA: Associated Professors at Karolinska Institutet, Sweden. |
| 4. | Sex | EM, AHP: Female; EA: Male |
| 5. | Experience and training | EM: Educational background in Occupational Therapy and Clinical Medical Science with practical experience in clinical Occupational Therapy work in both stroke unit in hospital and elder care in community healthcare setting. Colleague at the Faculty of Health and Occupational studies and Department of Public Health at University of Gävle and PhD student at the school of Health science at Karolinska Institutet, Division of Occupational Therapy. Experiences of qualitative data inquiry since 2014 as research assistant in Public Health research in Swedish community and municipality setting, as well as teaching qualitative methods, prevention and health promotion at bachelor and master program in Public Health science and Sustainable development.  AHP: Educational background in Occupational Therapy, practical experience of Occupational Therapy research and teaching, clinical work and quantitative and qualitative research.  EA: Educational background in Occupational Science, practical experience of Occupational Therapy research and teaching, clinical work and extensive experience in conducting qualitative research as well as teaching qualitative methods and methodologies at research school level. |
| *Relationship with participants* | | |
| 6. | Relationship established | Participants of the Make My Day stroke prevention programme |
| 7. | Participant knowledge of facilitator | Participants knew EM as researcher at Karolinska Institutet and interventionist of the Make My Day stroke prevention programme. |
| 8. | Facilitator characteristics |  |
| **Domain 2: Study design** | | |
| *Theoretical framework* | | |
| 9. | Methodological orientation and theory | Constructive grounded theory and a constant comparative method according to Charmaz [2]. |
| *Participant selection* | | |
| 10. | Sampling | Recruiting from Make My Day stroke prevention programme piloted in different suburbs in Region Stockholm, Sweden [3]. The Make My Day prevention programme is situated in Swedish primary healthcare setting. |
| 11. | Method of approach | Telephone contact and information sent out by email. |
| 12. | Sample size | 13 |
| 13. | Non-participation | 1 (one that declined participation after invitation) |
| *Setting* | | |
| 14. | Setting of data collection | Quiet locations agreed upon with the participant (conference room at university, room at the primary healthcare facility, local café) |
| 15. | Presence of non-participants | No |
| 16. | Description of sample | Participants consisted of six men and six women aged 49–70 years with a mean age of 59 years, recruited from the city or suburbs of Stockholm, in Sweden. All participants had access to either a smartphone or other wireless device and all but one participant were frequent users of smart phone technology in everyday life, however, to different extent for self-monitoring of health before participation in Make My Day. Eight participants perceived that they had the skills needed for using smart phone technology and different health apps, while four participants did not. All participants had moderate to high risk for stroke (3-6 risk factors in total), albeit different modifiable and/or non-modifiable risk factors. Six participants had in addition to the different modifiable risk factor for stroke also experienced a Transient Ischemic Attack prior to their participation in the Make My Day prevention programme, however, none of the participant had had a previous stroke. |
| *Data collection* | | |
| 17. | Interview guide | Interview guide produced within the larger research group consisting of researchers with a background in Medicine, Occupational Therapy, Occupational Science and Public Health, after reviewing relevant empirical and methodological literature [example of literature: 4-15]. Overarching topics and some specific questions are provided in the methods section for each group of participants. The interview guide can be translated to English and shared upon request. |
| 18. | Repeat interviews | Two pilot interviews and observations were performed (not included in the study). One person with medical expertise from the research team and one external expert person in digital technology were interviewed and observed. These interviews and observations yielded feedback that was helpful in developing the interview guides to ensure open-ended questions and to train the observer to stimulate participants to verbally reflect on what they were doing and experiencing in the moment, when using the app during the observations. After pilot interviews had been conducted interviews were conducted with study participants, where some of the participants were interviewed at several occasions. |
| 19. | Audio/visual recording | Audio recording |
| 20. | Field notes | Field notes were written during the data gathering as well as observational notes during the interviews and observations, and both was included in the analysis. |
| 21. | Duration | 18 months (June 2018 - February 2020) |
| 22. | Data saturation | Yes |
| 23. | Transcripts returned | Transcripts were not returned to the participants. Questions for validating answers were asked at each interviews. For the persons where repeated interviews were done, previous topics and answers was followed up. |
| **Domain 3: Analysis and findings** | | |
| 24. | Number of coders | Three (EM, AHP, EA). |
| 25. | Descriptions of coding tree | A description of the analysis process is presented in the manuscript along with examples of initial codes, focused codes and frequent codes that memos were written about. In the result section the final themes, subthemes, quotes, and a conceptual figure interlinking findings are presented to provide insight into the findings and process. |
| 26. | Derivation of themes | Inductive coding |
| 27. | Software | Atlas.ti |
| 28. | Participant checking | No |
| *Reporting* | | |
| 29. | Quotations presented | Yes |
| 30. | Data and findings consistent | Yes |
| 31. | Clarity of major themes | Yes |
| 32. | Clarity of minor themes | Yes |

1. Tong A, Sainsbury P, Craig J. Consolidated criteria for reporting qualitative research (COREQ):a 32-item checklist for interviews and focus groups. International Journal for Quality in Health Care.2007;19(6):349-357.
2. Charmaz K. *Constructing grounded theory*. 2nd ed. London, Thousand Oaks (CA): SAGE Publication Ltd., 2014.
3. Patomella A-H, Guidetti S, Mälstam E, et al. Primary prevention of stroke: Randomised controlled pilot trial protocol on engaging everyday activities promoting health. *BMJ Open* 2019; 9: e031986.

Kvale, S and Brinkmann, S. *Interviews: Learning the craft of qualitative research interviewing*. 3rd ed. London, Thousand Oaks (CA): Sage Publications, 2015.

1. Charters E. The use of think-aloud methods in qualitative research. An introduction to think-aloud methods. *Brock Education* 2003; 12: 68–81.
2. Wilcock A and Hocking, C. *An occupational perspective of health.* 3rd ed. USA, Thorofore: SLACK Incorporated, 2015.

Brandt CJ, Clemensen J, Nielsen JB, et al. Drivers for successful long-term lifestyle change, the role of e-health: A qualitative interview study. *BMJ Open* 2018; 8: e017466. doi:10.1136/ bmjopen-2017-017466

Akenine U, Barbera M, Beishuizen CRL, et al. Attitudes of at-risk older adults about prevention of cardiovascular disease and dementia using eHealth: A qualitative study in a European context. *BMJ Open* 2020; 10: e037050.

Bartels SL, van Knippenberg R JM, Dassen F CM, et al. A narrative synthesis review of digital self-monitoring interventions for middle-aged and older adults. *Internet Interventions* 2019: 18, 100283.

1. Widmer RJ, Collins NM, Collins CS, et al. Digital health interventions for the prevention of cardiovascular disease: A systematic review and meta-analysis. *Mayo Clin Proc* 2015; 90: 469–480.
2. Michie S, Richardson M, Johnston M, et al. The behavior change technique taxonomy (v1) of 93 hierarchically clustered techniques: Building an international consensus for the reporting of behavior change interventions. *Ann Behav Med* 2013; 46: 81–95.
3. Klimis H, Thakkar J, Chow CK. Breaking barriers: Mobile health interventions for cardiovascular disease. *Can J Cardiology* 2018; 34: 905–913.
4. Krah E, Kruijf J. Exploring the ambivalent evidence base of mobile health (mHealth): A systematic literature review on the use of mobile phones for the improvement of community health in Africa. *Digital Health* 2016; 2: 1–20.
5. Alessa T, Abdi S, de Witte, L. Mobile apps to support the self-management of hypertension: Systematic review of effectiveness, usability and user satisfaction. *JMIR mHealth and uHealth* 2018; 6: e10723.
6. Silva BM, Rodroguea JJ, de la Torre Diez I, et al. Mobilehealth: A review of current state in 2015. *J Biomed Inform* 2015; 56: 265–272.
